# A lifestyle-based needs assessment of two European communities prior to the development and deployment of a digital health coaching smartphone application

**DOI:** 10.3389/fpubh.2025.1673205

**Published:** 2025-10-30

**Authors:** Marina Iglesias-Cans, Croia Loughnane, Roisin O’Donovan, Ciprian Iftimoaei, Diana Vasiliu, Manuela Iftimoaei, Ingrid Enache, Angela Achitei, Padraic J. Dunne

**Affiliations:** 1 Centre for Positive Health Sciences, RCSI University of Medicine and Health Sciences, Dublin, Ireland; 2 Department of Political Science, Faculty of Law, Economics, Political and Administrative Sciences, “Petre Andrei” University of Iași, Iași, Romania; 3 National Institute for Research & Development in Informatics – ICI, Bucharest, Romania; 4 ADV, Alături de Voi Foundation, Iași, Romania; 5 Research Institute for Quality of Life – Romanian Academy, Bucharest, Romania

**Keywords:** positive health sciences, positive health coaching, digital health interventions, health promotion, disease prevention, needs assessment, community-based participatory research, lifestyle medicine

## Abstract

**Introduction:**

There is growing interest and demand for leveraging digital technology to enhance the reach and scalability of health and wellbeing interventions, as a solution to the global burden of noncommunicable diseases. For positive health sciences, digital technology has been identified as a feasible solution to increasing the accessibility, scalability and reach of positive health interventions. While digital health solutions have the potential to improve the health and wellbeing of communities and reduce the burden on health systems, these solutions often take a one-size-fits-all approach in their design and implementation. This study aimed to conduct a Community-Based Participatory Research (CBPR) informed needs assessment in Athy (Ireland) and Iași (Romania) to identify lifestyle-related health challenges, barriers, and digital engagement preferences in preparation for implementing the Connect5 digital health coaching intervention.

**Methods:**

A mixed-methods cross-sectional design was employed, involving quantitative surveys (*n* = 219 in Ireland; *n* = 205 in Romania) and qualitative interviews/focus groups. Surveys assessed sociodemographic characteristics, lifestyle habits, barriers, support needs, and digital health perspectives.

**Results:**

Both communities reported challenges with sleep, physical activity, sedentary behavior, and stress, with common barriers including lack of time (due to work/study/childcare) and the high cost of healthy food. Notable variations in lifestyle needs, barriers, and support preferences were observed across sociodemographic groups within each community, including gender, age, education, and residency. Lower socioeconomic groups faced more systemic barriers like high food costs or lack of safe outdoor spaces. Despite different levels of prior engagement with digital solutions, both communities showed strong interest in digital health coaching solutions.

**Discussion:**

The findings from this needs assessment will inform the design and implementation of the upcoming Connect5 project and provide broader insights for future digital health initiatives. They highlight the importance of integrating community voices and sociodemographic insights to ensure that digital health solutions are relevant, inclusive, and equitable, ultimately promoting sustained engagement, reducing health disparities, and improving population wellbeing.

## 1 Introduction

Leveraging digital technologies is vital for achieving universal health coverage, enhancing health promotion, and serving disadvantaged populations (1). Moreover, digital technology offers a promising solution to combat the global burden of noncommunicable disease (NCDs) (2–4), which are responsible for 41 million deaths annually, accounting for 70% of all global deaths, and 40% of premature deaths among individuals under 70 years (5, 6). NCDs such as cardiovascular disease, type-2 diabetes mellitus, chronic respiratory diseases and certain cancers are largely preventable through the elimination of lifestyle risk factors like tobacco use, poor nutrition, low physical activity, and alcohol use (5, 7, 8), making health promotion interventions particularly effective for tackling the global NCD burden. Despite significant investments and advancements in health promotion interventions, progress has been slow, with only a 2% global reduction in premature deaths recorded between 2010 and 2019 (9). This limited progress may be partly due to the scalability and accessibility constraints of traditional health promotion efforts, especially when targeting hard-to-reach and vulnerable populations (10–12).

Digital technologies can help overcome these limitations by providing scalable, accessible, cost-effective, and equitable health promotion and disease prevention interventions (1, 13). The integration of digital technologies across various sectors of health and wellbeing is catalyzing transformative change toward more accessible and equitable services that address the NCD burden at a lower cost (14–16). Recognized by the World Health Organization (WHO) as a key enabler of the Sustainable Development Goals (1, 3), this transformative shift toward digital health has sparked a digital revolution within healthcare systems (17). Digital health technologies, like smartphone applications and wearable devices, are gaining popularity and show strong potential for supporting lifestyle changes and chronic disease management (4, 18–20). Key advantages include personalized real-time support, convenience, accessibility, broad reach, scalability, security, efficiency, effortless access to evidence-based educational materials, and proven efficacy (18). Together, these innovations present a powerful opportunity to transform how health promotion, prevention, and chronic disease management are delivered. Notably, one emerging field that stands to benefit significantly from these digital advancements is positive health sciences; a holistic and multidimensional field of research which takes the care continuum past the point of absence of disease and focuses on the knowledge, skills, and empowerment required for individuals and communities to thrive (21).

Positive Health Sciences (PHS) is deeply rooted in the theoretical foundations of positive psychology (22), lifestyle medicine (23), and health psychology (21, 24, 25). PHS is also grounded in the emerging concept of meliotropism that describes the dynamic orientation and tendency of human beings toward meaningful, prosocial, health-promoting actions that benefit individuals and communities alike (26). PHS provides a range of positive health interventions that aim to build a person’s meliotropic orientation toward positive health; most notably, positive health coaching (PHC). PHC has been developed to effectively translate positive health science and research into a feasible intervention that can support individuals to make changes for better health (27, 28). However, like many health promotion and disease prevention approaches, PHC faces challenges related to scalability and accessibility. In response, researchers are increasingly exploring digital technologies as a means to broaden the reach and impact of positive health interventions.

We have previously tested the integration of digital technologies and PHS through a coach-led digital positive health intervention for Irish-based hospital workers during the COVID-19 pandemic, called RCSI Coach Connect (25, 29, 30). This digital health solution yielded positive engagement, improved health (related to the pillars of lifestyle medicine), and mitigated burnout among 23 hospital workers (29). Building on the feasibility of this positive health intervention, we are now in the process of developing a digital initiative for community implementation that we call Connect5.

The Connect5 project is a community-based study that aims to effectively support positive health changes in communities by implementing a human coach-led digital health solution. We hope to implement PHC in a way that is both scalable, accessible and meaningful to the community. This solution will be designed using the theoretical foundations of PHS and delivered through PHC via a digital health smartphone application. However, researchers have highlighted the limitations and risks of adopting a one-size-fits-all approach to digital interventions (31–33). They emphasize the importance of accounting for the distinct socioeconomic and environmental factors that shape health and wellbeing in each community, and the need to embed these considerations into the design of community-based interventions (32–34).

Digital health provision can both revolutionize the delivery of health interventions, empower individuals and promote agency, but also risks isolating and excluding certain populations and groups (32, 33, 35, 36). The WHO (2021) has highlighted that while digital technologies are rapidly advancing to improve access to care, this is not sustainable or equitable across all populations and groups. Digitally excluded groups include older adults, socioeconomically disadvantaged populations, rural communities, ethnic and racial minority groups, and individuals with low digital literacy (32, 35–37). There is a clear digital divide that is crucial to bridge to ensure equitable and meaningful access to digital health (1). Research has highlighted the importance of contextually sensitive digital health technology as a solution for bridging this divide (33, 38). This approach advocates for the move away from a techno-deterministic design focus to one that is more contextually informed. Contextually sensitive technology essentially considers sociocultural factors in the design of digital health interventions to ensure technology is appropriate for communities and is meeting their specific health needs (33).

To address these challenges in digital health, a Community-Based Participatory Research (CBPR) informed approach to project design and implementation will be adopted for the design and implementation of Connect5. CBPR is a method of research that places vital community stakeholders at the center of research, using their experiential knowledge to co-create the research framework and health interventions alongside researchers and other health experts, where all stakeholders carry equal weighting (39–41). CBPR also emphasizes viewing the community as a dynamic social entity, rather than just a research cohort (40, 41). This approach provides a framework for understanding how these dynamic social entities, influenced by its specific socioeconomic and environmental determinants, defines health priorities and needs. As such, allowing researchers to co-design health promotion and prevention interventions according to the context and preferences of the targeted communities.

To deliver a digital health intervention tailored to the needs of the Connect5 target communities, this study conducted a parallel CBPR-informed needs assessment in Athy, Co. Kildare (Ireland) and Iași (Romania). The objectives were to explore community members’ health and wellbeing habits, needs, and barriers, and to gather perspectives on digital technology and positive health coaching to inform the implementation of the Connect5 intervention at each site. Based on the literature, the following research questions guided the assessment:

1. What are the primary health and wellbeing challenges and unmet needs in each target community?
2. What are community members’ current levels of engagement with, and attitudes toward, digital health technologies?
3. How do community members perceive positive health coaching, and what preferences do they have for coaching delivery methods?

## 2 Materials and methods

### 2.1 Setting

Two communities were selected for this study: Athy in Ireland, and Iasi in Romania. Athy is a rural community with a population of 10,837 people according to the 2022 National census (Central Statistics Office, 2022). Iași on the other hand, is the fourth largest city in Romania, the metropolitan area including Iași city plus 19 surrounding communes has a population of 423,154 according to the 2021 Romanian national census (National Statistics Institute, 2021). The Irish needs assessment was conducted by the Centre of Positive Health Science at the Royal College of Surgeons in Ireland (RCSI) University of Medicine and Health Sciences, in conjunction with Kildare County Council and local Sláintecare Healthy Communities supports. The Romanian needs assessment was conducted by researchers working for Fundația Alături de Voi (ADV Romania), a charity organization that promotes social entrepreneurship among Romanian communities.

### 2.2 Ethics

Ethical approval for this study was obtained from the RCSI Research Ethics Committee, Dublin (Reference number: REC202210030). Written informed consent was obtained from all respondents prior to taking part in the survey. Romanian respondents consented to the use of their anonymous data for research purposes as outlined by ADV Romania. In order to maintain anonymity, no identifiable information was collected during the survey data collection. Participants in interviews and focus groups were pseudonymized rather than anonymized to allow for data linkage and analysis while protecting their identities.

### 2.3 Study design

A quantitative cross-sectional study was used to assess the needs of residents in Athy in Ireland and Iași in Romania.

#### 2.3.1 Quantitative needs assessment survey

A cross-sectional needs assessment survey was conducted to explore the health and lifestyle needs of individuals living, working, or engaging in activities in Athy, Ireland, and the metropolitan area of Iași, Romania. The survey was developed by the research team at the RCSI Centre for Positive Health Sciences in English and translated to Romanian by ADV Romania (Supplementary Table S9). The survey was developed as an exploratory tool to gather informal insights into lifestyle habits, barriers, and support needs, and was not intended or designed as a validated psychometric instrument. The Romanian version was translated by a bilingual subject expert and reviewed for clarity and cultural appropriateness, but no formal validation was performed, as the study did not aim to conduct country-to-country comparisons. The survey in both communities aimed to assess respondents’ socio-demographic characteristics, lifestyle habits, needs, and behaviors, structured around the pillars of lifestyle medicine: sleep, nutrition, physical activity, stress management, substance use, and social relationships, with the addition of meaning and purpose in life. In 2019, researchers reported the results of a cohort study of 6,985 American adults, which described how purpose in life was significantly associated with longer life- and health-span (42). This survey also used existing literature on standards of health according to these pillars to provide context for participants completing the assessment. For example, the recommended target for sleep was 7–8 hours per night (43), and the target for physical activity was 150 min of moderate-intensity activity (44). We also sought to gather insights into respondents’ engagement with technology, their perspectives on digital health solutions, and their interest in PHC. The questionnaire included both closed-ended questions, covering demographic background, lifestyle behaviors and needs, barriers to lifestyle changes and preferences for a smartphone-based health intervention. Open-ended questions were used to gather other barriers and solutions to lifestyle changes participants may have that were not in the predefined list.

### 2.4 Sample and recruitment

Residents of Athy and Iași who were over the age of 18 were invited to take part in the quantitative survey and qualitative interviews or focus groups. The Irish survey was disseminated by researchers at the Centre of Positive Health Science at RCSI, both online using secure Microsoft (MS) Forms and in hard copy at the local library and other local resource offices in Athy associated with Sláintecare Healthy Communities and Kildare County Council. The dissemination process was conducted in collaboration with officers and healthcare workers based at these two agencies in Ireland. To further broaden the reach, local online advertisement through Facebook was also used in Athy to target community members and to capture residents who may not be engaged with existing community support systems. The paid Facebook campaign was restricted to adults over 18 years residing and/or working in Athy and the surrounding hinterland (up to 5 kilometers).

The anonymous needs assessment survey was simultaneously distributed in Iași, Romania by the research group embedded within AVD Romania. The needs assessment was distributed online only through email or WhatsApp groups to the network of collaborators at AVD Romania and the Coalition of Organizations of Patients with Chronic Diseases in Romania (COPAC). Respondents were among the beneficiaries or clients of these collaborating institutions and non-governmental organizations (NGOs), as well as among their family members or friends.

### 2.5 Data collection and analysis

Anonymous survey data were collected in 2023 from completed online (MS Forms) or paper surveys distributed in both communities. Survey data were compiled in MS Excel, cleaned and analyzed using Stata 18.0 (45) and SPSS (46). All 59 categorical variables (5 sociodemographic variables, 12 lifestyle habits variables, 17 lifestyle barriers variables, 10 support with lifestyle variables and 15 digital health and coaching variables) were converted from string to numerical format, and descriptive statistics (frequencies and percentages) were calculated. For analysis, questions with more than three response options were grouped into two categories. For example, responses to the question with options “never, rarely, sometimes, often, and always” were consolidated into “never or rarely” and “sometimes, often, and always.” Additionally, for “yes,” “no,” and “I do not know” questions, if “I do not know” responses accounted for less than 5% of the total, they were recorded as empty cells. A global chi-square test of association was conducted to assess potential differences across sociodemographic groups in relation to lifestyle habits, lifestyle barriers, support needs, and perspectives on digital health coaching within each community.

It is important to note that given the differences in sampling, demographics, and cultural context, direct statistical comparisons between the Romanian and Irish communities were not feasible. Importantly, the primary aim of the study was to assess community-specific needs to inform targeted intervention design, rather than to conduct cross-community comparisons. Cross-country findings are not intended for direct comparison, but rather to highlight context-specific insights.

## 3 Results

### 3.1 Demographics

The Romanian and Irish surveys had 205 and 219 respondents, respectively. The socio-demographic characteristics of both communities are described in Table 1.

**Table 1:** Socio-demographic profile of participants in the study from Ireland (Athy) and Romania (Iasi).

	Ireland		Romania	
	(*N* = 219)		(*N* = 205)	
	Size (*n*)	Percentage (*%*)	Size (*n*)	Percentage (*%*)
Sex				
Female	156	71.23	136	66.34
Male	63	28.77	69	33.66
Age*				
Under 40	37	16.89	106	51.71
40 or over	180	82.19	99	48.29
Highest level of education*				
VE	31	14.22	20	9.76
SE	39	17.89	46	22.44
TE	143	65.6	139	67.8
Residency*				
SH	10	4.72	12	6.25
R	29	13.68	43	22.4
HO	171	80.66	139	71.35
Vulnerable groups*				
None reported	157	71.69	35	17.07
Living alone	25	11.42	20	9.76
Single parents	14	6.39	14	6.83
Remote workers	17	7.76	27	13.17
Perinatal people	4	1.83	16	7.8
Migrant	3	1.37	11	5.37

VE, vocational education; SE, secondary education; TE, tertiary education; SH, social housing or emergency accommodation; R, renting; HO, home owner. *Due to some missing data, both communities *n* size varies slightly across variables.

#### 3.1.1 Irish respondents

A total of 219 respondents completed the Irish questionnaire (Table 1). The majority of respondents were female (71%), aged 40 years or older (82%). A large proportion of respondents had attended third-level education (66%), while 18% had attended secondary school and 14% vocational school as the highest level of educational attainment. Eighty percent of respondents were homeowners, with a small proportion renting (14%) or living in social housing or emergency accommodation (5%). Finally, 28% of respondents reported belonging to one or more vulnerable groups (i.e., living alone, single parent, remote workers, perinatal people, or migrants).

#### 3.1.2 Romanian respondents

Two hundred and five Iasi residents completed the Romanian questionnaire (Table 1). The majority of respondents were female (66%). The representation of respondents over and under 40 years old was marginally different (48 and 52%, respectively). In terms of the highest level of education attained, 68% of respondents had attended third-level education, 22% attended secondary school, and 10% attended vocational school. Seventy-one percent of respondents were homeowners, 21% of the sample rented, and 6% were living in social housing or emergency accommodation. Finally, 83% of respondents reported belonging to one or more vulnerable groups.

### 3.2 Lifestyle-related findings

The needs assessment survey delivered in Athy, Ireland, and Iași, Romania, collected data to better understand the specific health and wellbeing needs of the two communities. Lifestyle-related findings provided insights into the percentage of respondents who meet the recommended guidelines of sleep, physical activity, healthy eating, social relationships, meaning and purpose in life, stress management, and substance use (Table 2). These findings also highlighted the barriers to engaging with lifestyle behaviors of the above areas, and what support is needed within the two communities (Tables 3, 4 respectively).

**Table 2:** Lifestyle habits and concerns related to the pillars of lifestyle medicine of participants in both communities, Ireland (Athy) and Romania (Iasi).

	Ireland		Romania	
	(*N* = 219)		(*N* = 205)	
	Size (*n*)	Percentage (*%*)	Size (*n*)	Percentage (*%*)
Quality sleep*				
Achieve the recommended hours sleep (7–9 h)?	(*n* = 215)		(*n* = 198)	
No	131	60.93	117	59.09
Yes	84	39.07	81	40.91
Physical activity*				
Do you think you have been getting enough exercise in the last 3 months?				
Weekly physical activity target (150 min/week)	(*n* = 210)		(*n* = 196)	
No	117	55.71	126	64.29
Yes	93	44.29	70	35.71
Sedentary behavior	(*n* = 217)		(*n* = 205)	
<4 h	91	41.94	92	44.88
>4 h	126	58.06	113	55.12
Eating healthy*				
Are you concerned about your eating habits?	(*n* = 218)		(*n* = 205)	
not at all or slightly concerned	137	62.84	63	30.73
somewhat or very concerned	81	37.16	142	69.27
How confident are you in relation to cooking healthy?	(*n* = 217)		(*n* = 205)	
not at all, low or neutral	72	33.18	72	35.12
high confidence	145	66.82	133	64.88
Positive social connections*				
Are strong relationships important for health?	(*n* = 215)		(*n* = 202)	
no	4	1.86	2	0.99
yes	211	98.14	200	99.01
Do you feel lonely?	(*n* = 217)		(*n* = 204)	
never or rarely	104	47.93	43	21.08
sometimes, often or always	113	52.07	161	78.92
Meaning and purpose*				
Is meaning and purpose important for health?	(*n* = 213)		(*n* = 202)	
No	10	4.69	4	1.98
Yes	203	95.31	198	98.02
Stress*				
Do you experience stress at home or work?	(*n* = 214)		(*n* = 189)	
No	77	36.49	66	34.92
Yes	134	63.51	123	65.08
How is your mental health?	(*n* = 219)		(*n* = 205)	
Poor or fair	91	41.55	68	33.17
Good, very good or excellent	128	58.45	137	66.83
Use of risky substances*				
Is alcohol consumption a problem for you?	(*n* = 216)		(*n* = 205)	
not applicable/I do not know	42	19.44	37	18.05
No	137	63.43	147	71.71
Yes	37	17.13	21	10.24
Do you smoke tobacco?	(*n* = 151)		(*n* = 205)	
Not applicable/I do not know	78	51.66	58	28.29
No	50	33.11	100	48.78
Yes	23	15.23	47	22.93

*Due to some missing data, both communities *n* size varies slightly across variables.

**Table 3:** Supports required by both communities, Athy in Ireland and Iasi in Romania, related to the pillars of lifestyle medicine.

Would you like support with any of the following?	Ireland		Romania	
	(*N* = 219)		(*N* = 205)	
	Size (*n*)	Percentage (*%*)	Size (*n*)	Percentage (*%*)
Sleeping*	(*n* = 215)		(*n* = 205)	
I do not know	39	18.14	18	8.78
No	61	28.37	39	19.02
Yes	115	53.49	148	72.2
Physical activity*	(*n* = 215)		(*n* = 205)	
I do not know	31	14.42	24	11.71
No	55	25.58	45	21.95
Yes	129	60	136	66.34
Healthy eating*	(*n* = 218)		(*n* = 205)	
I do not know	19	8.72	27	13.17
No	71	32.57	35	17.07
Yes	128	58.72	143	69.76
Choosing healthy foods	(*n* = 219)		(*n* = 205)	
I do not know	23	10.5	20	9.76
No	76	34.7	39	19.02
Yes	120	54.79	146	71.22
Creating meaningful relationships*	(*n* = 217)		(*n* = 205)	
I do not know	27	12.44	18	8.78
No	107	49.31	64	31.22
Yes	83	38.25	123	60
Connecting with people*	(*n* = 215)		(*n* = 205)	
I do not know	37	17.21	22	10.73
No	99	46.05	70	34.156
Yes	79	36.74	113	55.12
Finding meaning and purpose*	(*n* = 187)		(*n* = 205)	
I do not know	26	11.98	25	12.2
No	96	44.24	73	35.61
Yes	65	43.78	107	52.2
Managing stress*	(*n* = 219)		(*n* = 205)	
I do not know	23	10.5	15	7.32
No	67	30.59	40	19.51
Yes	129	58.9	150	73.17
Improving your mental health	(*n* = 219)		(*n* = 205)	
I do not know	26	11.87	15	7.32
No	68	31.05	51	24.88
Yes	125	57.08	139	67.8
Alcohol and/or tobacco use*	(*n* = 213)		(*n* = 205)	
I do not know	10	4.69	10	4.88
No	169	79.34	148	72.2
Yes	34	15.96	47	22.93

*Due to some missing data, both communities *n* size varies slightly across variables.

**Table 4:** Perspectives on digital health technology and Positive Health Coaching among participants from both communities, Ireland (Athy) and Romania (Iasi).

	Ireland		Romania	
	(*N* = 218)		(*N* = 205)	
	Size (*n*)	Percentage (*%*)	Size (*n*)	Percentage (*%*)
Have you ever used a smartphone app for health and wellbeing?*	(*n* = 214)		(*n* = 203)	
No	106	49.53	132	65.02
Yes	108	50.47	71	34.98
Have you ever used a coach to help with your health either online or in person?*	(*n* = 216)		(*n* = 205)	
No	162	75	155	75.61
Yes	54	25	50	24.39
Would you use a smartphone app for health and wellbeing that was developed specifically for you and your community in Athy?*	(*n* = 218)		(*n* = 204)	
I do not know	26	11.93	26	12.75
No	25	11.47	16	7.84
Yes	167	76.61	162	79.41
Would you use a smartphone app that had a real person (a coach) connected to it?*	(*n* = 218)		(*n* = 205)	
I do not know	37	16.97	14	6.83
No	18	8.26	21	10.24
Yes	163	74.77	170	82.93
Do you own a wearable device?*	(*n* = 216)		(*n* = 203)	
No	110	50.93	137	67.49
Yes	106	49.07	66	32.51
This new smartphone app will be able to connect and talk to wearable devices. Would this encourage you to use this app?*	(*n* = 217)		(*n* = 205)	
I do not know	39	17.97	45	21.95
No	29	13.36	36	17.56
Yes	149	68.66	124	60.49
Below you can see what this new smartphone app might be able to do for you. How likely would be to use this part of the app, if it was available to you?*				
Daily exercise programs	(*n* = 213)		(*n* = 205)	
Definitely not, unlikely or not sure	63	29.58	36	17.56
Definitely or likely	150	70.42	169	82.44
Videos on how to manage stress and mental health	(*n* = 169)		(*n* = 204)	
Definitely not, unlikely or not sure	47	27.81	40	19.61
Definitely or likely	122	72.19	164	80.39
An online program on The Science of Health and Happiness	(*n* = 163)		(*n* = 205)	
Definitely not, unlikely or not sure	60	36.81	39	19.02
Definitely or likely	103	63.19	166	80.98
Meditations for health and wellbeing	(*n* = 168)		(*n* = 204)	
Definitely not, unlikely or not sure	48	28.57	63	30.88
Definitely or likely	120	71.43	141	69.12
Meditations for sleep	(*n* = 165)		(*n* = 205)	
Definitely not, unlikely or not sure	51	30.91	66	32.2
Definitely or likely	114	69.09	139	67.8
Advice on food and cooking	(*n* = 157)		(*n* = 205)	
Definitely not, unlikely or not sure	36	22.93	29	14.15
Definitely or likely	121	77.07	176	85.85
If you had help using this app, would you be more likely to use it?*	(*n* = 217)		(*n* = 205)	
I do not know	45	20.74	26	12.68
No	37	17.05	23	11.22
Yes	135	62.21	156	76.1
Would you be interested in competing with other users in a team or individual setting using this new smartphone app?*	(*n* = 217)		(*n* = 205)	
I do not know	40	18.43	25	12.2
No	49	22.58	26	12.68
Yes	128	58.99	154	75.12
Would this type of competition motivate you to use this new smartphone app?*	(*n* = 216)		(*n* = 205)	
I do not know	44	20.37	45	21.95
No	44	20.37	42	20.49
Yes	128	59.26	118	57.56

*Due to some missing data, both communities *n* size varies slightly across variables.

#### 3.2.1 Athy, Ireland

Less than 40% of respondents from Athy achieved the recommended 7–9 h of sleep per night (Table 2). Over half (54%) did not meet physical activity targets, and 58% reported sedentary behavior of ≥4 h/day. In relation to nutrition and food habits, 63% were not concerned about their eating habits, though two-thirds (67%) expressed confidence in preparing healthy meals. Nearly all respondents valued relationships (98%) and having meaning and purpose (95%). However, 52% reported loneliness, 63% experienced stress at work or home, and only 58% rated their mental health as good to excellent. Seventeen percent consumed alcohol and 15% smoked tobacco.

Barriers to sleep, physical activity, and healthy eating were also assessed (Supplementary Table S10). Key barriers to physical activity included low motivation (53%), lack of time due to work/study (41%) or caring responsibilities (18%), in addition to open responses (*n* = 12) citing fatigue (33%) and chronic pain or chronic disease (33%). Suggested solutions (*n* = 101) included safer roads and public spaces (20%), and improved community initiatives and access to exercise groups (10%). For healthy eating, high food costs (40%) and lack of time (40%) were the main barriers, with additional challenges including household food preferences (23%) and limited knowledge (11%). Sleep was most often affected by stress and worry (67%), followed by work (38%) and screen use (30%).

When asked about supports (Table 3), respondents expressed the greatest need for help with physical activity (60%), healthy eating (59%), stress (59%), mental health (57%), and sleep (53%). Fewer respondents sought support with social connection (36–44%) or substance use (16%).

#### 3.2.2 Iași, Romania

In Iași, 41% of respondents achieved the recommended 7–9 h of sleep per night (Table 2). Most did not meet physical activity targets (61%) and over half (55%) reported ≥4 h/day of sedentary behavior. While 69% were concerned about their eating habits, 65% felt confident cooking healthy meals. Nearly all respondents valued relationships (99%) and purpose in life (98%). However, loneliness was common, with only 21% rarely or never experiencing it. Two-thirds reported stress at work/home (65%) and good to excellent mental health (67%). Finally, 10 and 23% of Romanian respondents consumed alcohol and smoked tobacco, respectively.

Like Athy, barriers to sleep, physical activity, and healthy eating were assessed (Table 3). Key barriers to physical activity included lack of time due to work/study (42%) or caring responsibilities (28%), and limited access to facilities (23%). Other challenges cited were lack of time (18%), and lack of adapted environments to exercise (16.5%). Suggested solutions included affordable gyms, community-based group activities, and accessible facilities for those with health conditions.

For healthy eating, the main barrier was cost (66%), followed by lack of time to cook (32%), taste preferences (14%), and limited knowledge (12%). Respondents also mentioned difficulties accessing healthy produce and managing portion sizes. Stress and worry (58%) were the most common barriers to sleep, followed by work (43%), childcare (27%), and screen use (16%), alongside health problems and environmental factors.

Support needs were high across domains, with respondents seeking help for stress (73%), sleep (72%), healthy eating (70–71%), physical activity (66%), and mental health (68%). More than half also desired support with relationships (60%) and purpose in life (52%). Demand for lifestyle support was greater overall in the Romanian sample compared to the Irish sample (Table 3).

### 3.3 Lifestyle habits, barriers and support needs according to sociodemographic groups

#### 3.3.1 Athy, Ireland

Gender and age differences were most pronounced in the Irish sample (Supplementary Tables S1–S3 for habits, barriers and required support respectively). Women were less likely to meet physical activity targets (61% vs. 37% of men, *p* = 0.004) and more often cited childcare as a barrier (23 and 7% respectively; *p* = 0.008). They also reported higher levels of loneliness (58% vs. 37%, *p* = 0.003) and stress (67% vs. 46%, *p* = 0.007).

Respondents under 40 were more likely to experience loneliness (76% vs. 47%, *p* = 0.002), while those over 40 had higher tobacco use (45% vs. 30%, *p* = 0.003). Younger participants reported lack of time due to work or study as a barrier to exercise, while older adults more often cited injury and disability.

By education and housing, mental health status and exercise barriers differed. Those in social housing reported poorer mental health (80% poor/fair vs. 39–48% in other groups, *p* = 0.031), and greater difficulty accessing safe outdoor spaces for exercise. In contrast, respondents with third-level education more often reported work or study as a barrier to exercise (Supplementary Table S2).

#### 3.3.2 Iași, Romania

Significant differences in lifestyle habits and barriers were observed across gender, education and residency groups among Romanian respondents (Supplementary Tables S4–S6 for habits, barriers and required support respectively).

Alcohol and tobacco consumption rates were higher among men compared to women (alcohol: *M* = 23%, *F* = 4%, *p* = 0.000; tobacco use: *M* = 36%, *F* = 16%, *p* = 0.004). Additionally, a higher proportion of men reported TV and phones as a barrier to getting adequate sleep, and food not being enjoyed by family as a barrier to healthy eating, compared to women (*M* = 18%, *F* = 7%, *p* = 0.002; *M* = 25%, *F* = 10%, *p* = 0.025). Whereas 33% of women reported higher rates of childcare and caring responsibilities as barriers to exercise, compared to 17% of men (*p* = 0.022).

Those with third-level education had higher levels of sedentary behavior (61%, *p* = 0.041) and higher rates of loneliness (84%, *p* = 0.031) but reported better mental health (72%, *p* = 0.036). They were also more likely to cite work/study as barriers to exercise and cooking, while those with vocational or secondary education more often reported food cost and taste as barriers to healthy eating. Similarly, those with secondary education also identified living arrangements as a barrier to sleep.

Housing strongly shaped lifestyle barriers. Social housing residents were least likely to meet physical activity targets (*p* = 0.028) and more often cited lack of safe outdoor spaces and embarrassment when exercising as the main barriers to exercise (58%, *p* = 0.003 and 41%, *p* = 0.002 respectively). More respondents in social housing also reported perceived food unpalatability as a barrier to healthy eating (49%, *p* = 0.009), and sleep disruptions due to living arrangements as a barrier to sleep (30%, *p* = 0.039). Alternatively, homeowners reported work as a barrier to sleep (50%, *p* = 0.026).

In terms of support needs, significant differences were observed among the gender and residency sociodemographic groups (Supplementary Table S6). Men expressed greater interest in substance use support (32%, *p* = 0.002). Residents of social or emergency housing and renters reported greater interest in support with sleep hygiene (*p* = 0.046) and mental health (*p* = 0.027); additionally, social housing and emergency housing residents expressed a need for support with social connections (*p* = 0.009), and substance use (*p* = 0.042).

### 3.4 Perspective on digital health technology and positive health coaching

Overall, both communities, Athy and Iași, responded positively to the role of digital technology and PHC in helping them achieve their health goals (Table 4).

#### 3.4.1 Athy, Ireland

In the Irish sample, half of respondents (50%) had never used a health or wellbeing smartphone app, and three-quarters (75%) had never engaged with a health coach. Despite this, most expressed interest in digital tools: 77% reported willingness to use a community-specific health and wellbeing app, and 75% welcomed integrated health coaching. Nearly half (49%) owned a wearable device, and 69% indicated that device connectivity would encourage app use. Interest in specific app features was high, with 70% likely to use daily exercise programs, 72% stress management videos, 71% meditation for health, 69% meditation for sleep, and 77% food and cooking advice. Support in using the app was seen as important, with 62% more likely to engage if guidance were provided. Gamification was also appealing, with 59% expressing interest and the same proportion reporting it would motivate regular use.

#### 3.4.2 Iași, Romania

In the Romanian sample, 65% of respondents had never used a smartphone health app and 76% had not engaged with a health coach. Nonetheless, most expressed interest in digital support: 79% were willing to use a community-specific health and wellbeing app, and 83% supported the integration of health coaching. Wearable ownership was lower than in Ireland, with 32% owning a device, but 60% reported that connectivity would encourage app use. Interest in app features was strong, with 82% likely to use daily exercise programs, 80% stress management videos, 69% meditation for health, 68% meditation for sleep, and 86% food and cooking advice. Engagement was also linked to support, with 76% more likely to use the app if guidance was provided. Gamification features were appealing to 75% of respondents, and 58% indicated that gamification would motivate use.

### 3.5 Perspective on digital health technology and positive health coaching according to sociodemographic groups

#### 3.5.1 Athy, Ireland

In Athy, Ireland, significant differences were observed in the education group (Supplementary Table S7). Respondents with a third-level education degree (TE) were significantly more likely to have used a health and wellbeing app (60%) and a health coach (32%) compared to those with vocational education (VE) (42 and 6%) and secondary education (SE) (26 and 13%) (*p* = 0.001 and *p* = 0.002 respectively). Additionally, respondents with third-level education were more likely to engage in meditations for health and well-being (80%) compared to respondents with vocational (50%) and secondary-level education (55%) (*p* = 0.002). No other significant differences were observed across gender, age, or residency groups.

#### 3.5.2 Iași, Romania

In Romania, significant differences in perspectives on digital health and coaching were observed across gender, age and education groups (Supplementary Table S8).

Women reported greater interest in specific digital health features compared with men. Seventy-five percent of women indicated willingness to use meditation for health and wellbeing and meditation for sleep, compared with 57 and 54% of men (*p* = 0.01 and *p* = 0.002). Cooking advice features were also more popular among women, with 90% expressing interest (*p* = 0.026).

Younger respondents (<40 years) reported higher prior engagement with digital health tools. Forty-five percent had used a smartphone health app compared to 24% of those over 40 (*p* = 0.002), and 31% had worked with a health coach compared to 17% of older respondents (*p* = 0.02). Wearable device ownership was also more common among younger adults (45% vs. 18%, *p* < 0.001).

Education level was associated with differences in wearable use and app preferences. Respondents with third-level education were more likely to own a wearable device (44%) compared with vocational (5%) and secondary-educated participants (9%) (*p* < 0.001), and more often indicated that device connectivity would encourage app use (72% vs. 30% vocational, 39% secondary; *p* < 0.001). In terms of app features, third-level and secondary-level respondents expressed greater interest in daily exercise programs (86 and 80%) compared with vocational (60%, *p* = 0.014). Similarly, interest in meditation for health and wellbeing was higher among third-level and secondary-educated respondents (75% each) compared with vocational (40%, *p* = 0.012). Meditation for sleep was also more popular among those with third-level education (74%) compared with vocational (45%) and secondary education (41%, *p* = 0.011).

## 4 Discussion

Digital health interventions (DHI) are often criticized for a lack of user involvement and cultural and contextual considerations in design and adoption, understanding technical support needs and considering the individual characteristics of end-users (33, 38). Understanding context and the specific needs of specific populations is critical for ensuring that DHIs are inclusive and accessible, particularly for vulnerable groups or digitally hesitant populations (38, 47, 48). This needs assessment was a critical step in informing the contextually sensitive design of a subsequent community-based DHI called Connect5.

The results of this study revealed interesting findings across, between, and within the two communities of Ireland and Romania. At a broader comparative level, the sociodemographic characteristics of the two communities exhibited some commonalities and differences. Respondents in Ireland and Romania were predominantly women and individuals with third-level education. However, differences emerged in age distribution and representation of vulnerable or disadvantaged groups. Ireland had an older sample, with the majority of respondents being over 40 years old, whereas Romania had a much higher representation of vulnerable or disadvantaged groups. These demographic commonalities and differences are important to consider when examining variations in health behaviors, perceived barriers, and support needs discussed later in this section.

Similarly, although lifestyle habits and barriers to healthy lifestyle behaviors appeared similar across the two communities, some differences emerged relating to barriers to healthier lifestyles and preferences for support. Both communities reported challenges with sleep, exercise, sedentary behavior, and stress. Common barriers to healthy lifestyle behaviors in both Ireland and Romania included: (1) physical activity - lack of time for exercise due to work, study, and childcare or caregiving responsibilities, with low motivation being a particularly significant barrier for Irish respondents; (2) consuming healthy food – high cost of food (especially in Romania), lack of time for cooking or preparing meals, and individual or family preferences for unhealthy foods; and (3) sleep - difficulty getting adequate sleep due to stress and worry in both communities, as well as work, children, and screen time. While work and children were more significant barriers to sleep in the Romanian community, the Irish community reported screen time as a more problematic factor. These barriers highlight the complexities individuals face in maintaining healthy habits across various aspects of their lives. Regarding support demand and preference, participants in Ireland most commonly expressed interest in support for physical activity, followed by healthy eating and food choices, then stress and mental health. In contrast, Romanian participants prioritized support for stress, followed by sleep, then healthy eating patterns and food choices.

Although both communities generally reported similar lifestyle behavior challenges and barriers, there was a notable contrast in their support needs and preferences. These findings highlight the pressing need to address gaps in community support systems and the requirement to empower individuals to build a strong foundation for a healthy lifestyle. These results also reflect the reality that communities are dynamic social entities, influenced by socioeconomic and environmental determinants which defines health priorities and the contextualized needs from community to community (40, 49). The dynamic social factors shaping health determinants and community priorities extended beyond the documented differences between communities. Interestingly, this study highlighted that health and wellbeing needs were not generalizable to the community as a whole. Instead, distinct patterns in health and wellbeing behaviors, barriers, and needs were observed across sociodemographic groups within the two communities.

### 4.1 Sociodemographic factors shaping health behavior, barriers and lifestyle support preferences

Notable differences in health behaviors, barriers, and support needs emerged across sociodemographic groups in Ireland and Romania. Both communities reported meaningful sociodemographic differences across gender and age groups, though these differences were associated with distinctly different outcomes. Additional sociodemographic differences in educational attainment were reported in Romania and residency status in Ireland.

#### 4.1.1 Gender

In both Ireland and Romania, gender differences significantly shaped health behaviors, barriers, and support needs. Women in Athy reported significantly more feelings of loneliness, higher stress levels, and lower physical activity than men. When asked about barriers to a healthy lifestyle, women in both communities faced significantly more barriers to exercise due to childcare and caring responsibilities, with the addition of stress as a barrier for women in Athy. Gender-specific lifestyle challenges were highlighted for men as well, specifically within the Romanian cohort. Men in Iași reported significantly higher alcohol and tobacco use compared to women, as well as a greater need for support with substance use.

These patterns reflect extensive evidence showing how gender disparities shape key determinants of health, including individuals’ motivation, opportunities, and barriers to healthy living. A wide body of research supports these findings, consistently showing that women are more likely to experience stress and social isolation, as well as childcare and caring responsibilities as barriers to engaging with healthier lifestyles (50–56). Contextually, our results also aligned with previous Irish studies showing that women in Ireland tend to be less active than men (57–61). In contrast, the higher levels of tobacco and alcohol use among men in Romania, echo consistent international findings indicating that men experience more smoke- and alcohol-related health problems and challenges (62, 63). Patterns of gender-based differences in lifestyle habits, barriers and support needs identified in this study and confirmed by the wider body of literature underscore the influence of deeply embedded social roles and responsibilities. These findings highlight the importance of gender-sensitive health promotion strategies that address the specific challenges and barriers women and men face within their communities.

#### 4.1.2 Age

Our needs assessment also found age-specific health needs across both communities, which were generally consistent with the existing scientific literature. In Ireland, respondents under 40 years reported significantly poorer mental health and greater loneliness. This finding aligns with evidence that younger adults often experience heightened psychological distress due to work, social and financial pressures, which usually decreases with age (64). Patterns of tobacco use also showed age-related differences in Ireland. Adults over 40 years reported significantly higher tobacco consumption than their younger counterparts, despite research suggesting that smoking rates are typically higher in adults under 40 (65, 66). This discrepancy may reflect lower cessation rates among older smokers, as highlighted by Fidler and colleagues (67), as well intensive governmental health promotion campaigns in Ireland, since 2005. While some progress in cessation is evident among older Irish adults, continued targeted interventions remain essential (68). However, the absence of similar or significant patterns of lifestyle habits in Romanian respondents suggests that cultural or contextual factors may influence age-related differences.

Despite this, age-specific barriers to health were reported in both settings. Time constraints due to work and study emerged as a prominent barrier to physical activity, particularly among Irish respondents under 40 years. This is consistent with research showing time as one of the most commonly cited obstacles to physical activity (69). Similarly, the younger Romanian cohort (under 40 years) exhibited significantly more barriers to healthy eating due to a lack of knowledge about healthy foods compared to the older cohort. Existing research has reported similar patterns of heightened sense of awareness and increased knowledge of healthy eating among older adults, suggesting knowledge grows with age (70, 71). Finally, among older adults in Athy, injury and disability were commonly reported as barriers to exercise, which reflects existing research linking physical limitations and fear of injury to reduced exercise participation in older populations (72).

Together, these patterns offer valuable insight into the distinct needs and challenges of younger and older cohorts in Ireland and Romania. The findings suggest that interventions promoting work–life balance and time management for physical activity, along with targeted nutrition education, would benefit younger adults in both communities. Meanwhile, older adults may require additional support and resources for managing injury-related barriers to exercise, as well as enhanced smoking cessation services tailored to their specific needs.

#### 4.1.3 Education levels

Respondents from lower socioeconomic groups were more affected by environmental and systemic health determinants, whereas higher socioeconomic groups experienced more individual-level barriers. This was particularly evident in Romania, where respondents with third-level education reported lack of time due to work and study commitments as a major barrier to exercise, and lack of time to cook as a barrier to healthy eating, significantly more than those with vocational or secondary level education. Similar patterns were observed in Ireland, where third-level educated respondents also reported time constraints as a key barrier. However, the Romanian data revealed additional nuances between education groups. Respondents with vocational or secondary education in Romania more frequently reported the high cost of healthy food as a barrier to healthy eating and living arrangements, and work (secondary level only) as a barrier to sleep. However, work also significantly impacted the sleep of respondents with third-level education.

Other barriers which disproportionately affected respondents with a maximum secondary or vocational educational attainment, included injury and disability as a barrier to exercise, and the perceived unpalatability of healthy food. Lifestyle habits also varied across education levels in Romania. Those with third-level education reported more sedentary behavior and greater experiences of loneliness, while those with vocational or secondary education reported higher stress levels and poorer mental health. These findings suggest lifestyle habits and barriers vary between education groups and may reflect deeper socioeconomic and structural inequalities. Our findings support existing research that demonstrates that social inequality and structural barriers exist in the ability of lower socioeconomic groups to stay healthy, even with cost-free and equal access to health care (73, 74). These barriers ultimately lead to lower socioeconomic groups facing greater challenges in adopting healthier lifestyles (74). However, it should be noted that while individual-level barrier was cited as more common among high socioeconomic groups, it’s important to recognize that even these barriers can be shaped broadly by societal pressures and expectations.

#### 4.1.4 Residency

Lifestyle habits, support needs, and barriers that correlated with the residency of respondents continue to underscore the critical role of systemic and environmental barriers uniquely experienced by individuals based on their socioeconomic status (73, 74). Residency correlated with similar trends to those observed by education level. Similar to respondents with third-level education, renters and homeowners also reported a lack of time as a barrier to exercise. Similarly, the perceived unpalatability of respondents or their family was a barrier to healthy eating for those living in social housing. Furthermore, their living arrangements negatively impacted sleep, much like respondents with secondary or vocational education as the highest level of educational attainment. One notable deviation was that work commitments impacted renters and homeowners, similar to how they affected those with vocational and secondary education. Additional findings relating to residency and lifestyle habits showed that those living in social housing in Ireland were disproportionately affected by poor mental health compared with the other groups.

Additional insights emerged when examining the support needs and barriers experienced by individuals in lower socioeconomic groups, such as social housing residents. While the subsample of Romanian respondents in social housing who did not meet recommended physical activity targets was small, these findings align with previous research reporting that lower socioeconomic groups tend to have significantly lower levels of physical activity (75). Furthermore, the present study found that social housing residents faced heightened barriers due to limited access to safe outdoor spaces and embarrassment about exercising, consistent with evidence linking environmental disadvantage and social stigma to lower physical activity engagement (76, 77). These barriers create significant obstacles to engaging in health-promoting behaviors. Patterns in support needs further reflect the social disadvantages associated with living in social housing. Significant correlations between support needs and residency status pointed to greater reported demand for support related to risky substance use, mental health, and sleep among Romanian social housing residents, while Irish respondents in social housing more frequently reported a need for support in connecting with other people. Significant correlations between support needs and residency indicated greater reported demand for support related to risky substance use, mental health, and sleep for Romanian respondents in social housing, whereas Irish residents more often identified a need for social connection support. These support trends echo existing studies on the long-term impact of housing insecurity on mental health and wellbeing (78, 79).

While the behaviors, preferences, and needs across and within communities of this needs assessment align with existing literature, it is evident from the results that generalizability is still not possible, nor should it be promoted. Different socioeconomic groups expressed different challenges and preferences reflecting complex intersections between individual, environmental, and social factors within and across Ireland and Romania. These findings reinforce that the one-size-fits-all approach is insufficient for digital health and community interventions (31). Instead, CBPR approaches like the one applied in this study are essential for identifying both community-wide and subgroup-specific needs. This ensures that health promotion and prevention interventions are designed according to the context and preferences of the targeted communities and the groups within it.

### 4.2 Digital health and PHC

Both communities showed strong interest in digital health solutions and PHC. Despite a large proportion of respondents having never engaged with a health app or health coaching, the majority of respondents across communities and sociodemographic groups were willing and interested in using a community-specific health and wellbeing app with a health coach. Likewise, the ownership of wearable devices like smartwatches (i.e., Garmin, Fitbit, and Apple Watch) was relatively low. However, a majority of respondents still reported an interest in the ability to connect wearable devices to the digital solution and its ability to motivate and encourage use of the DHI. These findings highlight the acceptability and potential for a coach-led digital positive health intervention to bridge gaps in health support, particularly for vulnerable groups. These levels of acceptability align with previous findings indicating a growing interest in health and well-being digital technology across various population groups (80).

However, it is important to note that younger individuals and those with higher education were more likely to have used health apps, engaged with a health coach, and owned wearable devices than older respondents and those with secondary or vocational education. This suggests that these services may typically be more accessible or catered to higher socioeconomic groups and younger digital generations, despite needs and preferences reported as higher in lower socioeconomic or marginalized groups. Although the purpose of digital health is to improve the availability and accessibility of services like health coaching to typically hard-to-reach populations, research has shown that this is often not the case; lower socioeconomic populations, people with migrant backgrounds, older adults, women, and negatively racialized groups are usually at risk of being marginalized or excluded from digital health interventions (81–83). Technology and DHI are not socially neutral. They are shaped by the context in which they are established (81). Results from the scientific literature have indicated that inequity in societies and healthcare systems is usually reflected or exacerbated in digital health applications and use (81–83). In addition, not everyone has equal access or opportunity to benefit from the digitalization of health services (83).

Additionally, barriers to access include inadequate skills (i.e., digital skills), lack of time, capacity, resources or confidence in the digital intervention, as well as broader political, social, and economic systemic factors that hinder engagement with DHIs (81, 83). To overcome this inequity, an intersectional approach to digital health is recommended, which considers barriers to engagement and social determinants of health in its design and delivery (82, 83). This has also been advised by WHO (2021) in their global strategy on digital health 2020–2025, where relevant factors of inequalities should be assessed to ensure that access for specific populations is guaranteed, and no one is left behind (84).

Regarding the specific DHI proposed for the subsequent Connect5 project, respondents showed a preference for gamification features, suggesting that incorporating gamification elements could enhance engagement. Respondents were also asked about different application features like exercise programs, stress management videos, food and cooking advice, and guided meditation. While all features were positively received by respondents, there were some differences in preferences according to sociodemographic groups. For example, in both countries, women and respondents with third-level and secondary-level education showed greater interest in meditation features compared to men and respondents with vocational education. Likewise, respondents with third level and secondary level education showed greater interest in daily exercise programs. While there were only slight differences in features preferred among different sociodemographic groups, these findings continue to support the overall hypothesis that digital health interventions should be tailored to the targeted community and sociodemographic groups that exist within the community for sustainable engagement and experience. This is supported by a systematic review by Lattie et al. (85) that identified that improving user experience and tailoring user engagement appeared vital for the sustainable implementation of digital mental health interventions. These barriers are in line with previous studies that identified key factors that would increase uptake and engagement of digital health solutions, including digital literacy and technical support, personalization, social networking, and gamification, endorsing the importance of tailored smartphone app features and engagement strategies (80).

Our needs assessment found that each of these factors was identified as important for sustaining engagement with the DHI. However, one other factor was identified in this needs assessment, the presence of a human health coach and human technological support on the digital platform. Respondents across both communities responded positively to the added feature of a human health coach on the digital application. Likewise, the addition of support in using the digital health technology and navigating the application seemed to correlate with respondents being more likely to engage with the intervention. A recent systematic review supports these findings (86). While AI and human coaches produced similar health and wellbeing outcomes, human coaches could build better relationships and rapport with participants, which in turn supported increased and sustainable engagement with the digital health interventions. Due to the strong sense of connection, warmth and authenticity of human coaching, participants generally reported a preference for the addition of human coaching in digital health interventions (86).

## 5 Strengths and limitations

The study’s strength lies in the use of a CBPR approach, prioritizing the voices of community members in co-designing digital health interventions. The research captures a broad range of health behaviors, barriers, and support preferences across two diverse communities. Additionally, it highlights the strong acceptability of digital health coaching among participants. By giving participants a central role in shaping the intervention, the study fosters greater community buy-in and supports the development of solutions that are culturally appropriate. This approach ensures that the digital health coaching intervention is not only relevant but also well-received, increasing its potential for adoption and sustained engagement within the communities.

We identified three main limitations in our study. First, despite efforts to recruit a diverse sample from the general population, the Irish cohort primarily consisted of middle-aged women (40 years or older), with low male participation (29%). A similar gender distribution was observed in the Romanian group; however, the age distribution was younger, with 52% of respondents under 40 years old. Nevertheless, the study provided valuable insights into health behaviors and digital health needs across different demographic groups. Sampling bias may have occurred in the Romanian cohort due to recruitment strategies through AVD Romania and the Coalition of Organizations of Patients with Chronic Diseases in Romania (COPAC). This recruitment method may have contributed to the Romanian group having a greater proportion (82%) of respondents belonging to disadvantaged groups and expressing a greater need for support. While this sample composition may have influenced the findings, it also highlights the needs of vulnerable groups, particularly those living with chronic illness, who require the most attention in health interventions. Second, the sample sizes within specific sociodemographic groups were limited, which reduced the statistical power of our descriptive and exploratory analyses. While the study provides valuable insights into lifestyle habits and support needs within these communities, we acknowledge that the findings should be interpreted with caution given the uneven representation of certain groups. Further research with larger, more balanced samples across sociodemographic groups is needed to strengthen the generalizability of these findings and provide a more comprehensive understanding of health needs in both communities. Finally, the reliance on self-reported data may have introduced response bias, as respondents might overestimate or underestimate their health behaviors, lifestyle habits, or support needs due to recall issues or social desirability.

## 6 Conclusion

Digitalizing positive health interventions has the potential to increase accessibility and scalability of health and wellbeing support across different communities. Such interventions can play a meaningful role in reducing the burden and prevalence of NCDs, aligning with the global UN goal of reducing NCD-related premature mortality rates. However, to ensure the effectiveness and relevance of DHIs for communities, it is vital to understand the socioeconomic and environmental determinants of health within targeted communities. This inclusive approach allows for the development of context-sensitive studies. This study employed a CBPR approach to assess the specific health and wellbeing needs of citizens in Athy, Ireland and Iași, Romania, ahead of the contextual adaptation and implementation of a subsequent project. Connect5, is a digital positive health coaching intervention, designed to promote greater health and wellbeing among community residents in both Athy and Iași.

The CBPR-informed needs assessments identified shared requirements for support in lifestyle areas such as sleep, physical activity, sedentary behavior, and stress management across both communities. Study respondents expressed strong interest in digital health coaching, particularly preferring interventions that combine human coaching with digital technology. The assessment revealed important variations in lifestyle needs and preferences not only between Romania and Ireland but also within sociodemographic subgroups based on gender, age, and socioeconomic status. These findings emphasize that effective DHIs require community engagement in a co-design process, integration of human support, and targeted approaches that address the specific needs of different community subgroups to promote health equity and improve population wellbeing.

### 6.1 Implications for future research

We encourage researchers and community support workers to move away from one-size-fits-all approaches when it comes to designing and delivering health interventions. Along with other researchers, we advocate the use of a bottom-up CBPR approach to tailor digital health solutions and reduce health inequities, especially at a community level. While this study included vulnerable populations, the sample was skewed toward higher socioeconomic backgrounds. Future research must prioritize reaching marginalized and digitally excluded groups to ensure their needs are fully reflected in equitable digital health solutions.

## Data Availability

The raw data supporting the conclusions of this article will be made available by the authors, without undue reservation.
