# Correction: A lifestyle-based needs assessment of two European communities prior to the development and deployment of a digital health coaching smartphone application

**DOI:** 10.3389/fpubh.2026.1804843

**Published:** 2026-03-04

**Authors:** Marina Iglesias-Cans, Croia Loughnane, Roisin O'Donovan, Ciprian Iftimoaei, Diana Vasiliu, Manuela Iftimoaei, Ingrid Enache, Angela Achitei, Padraic J. Dunne

**Affiliations:** 1 Centre for Positive Health Sciences, RCSI University of Medicine and Health Sciences, Dublin, Ireland; 2 Department of Political Science, Faculty of Law, Economics, Political and Administrative Sciences, “Petre Andrei” University of Iaşi, Iaşi, Romania; 3 National Institute for Research & Development in Informatics – ICI, Bucharest, Romania; 4 ADV, Alături de Voi Foundation, Iaşi, Romania; 5 Research Institute for Quality of Life – Romanian Academy, Bucharest, Romania

**Keywords:** positive health sciences, positive health coaching, digital health interventions, health promotion, disease prevention, needs assessment, community-based participatory research, lifestyle medicine

Lattie EG, Adkins EC, Winquist N, Stiles-Shields C, Wafford QE, Graham AK. Digital mental health interventions for depression, anxiety, and enhancement of psychological well-being among college students: systematic review. *J Med Internet Res*. (2019) 21:e12869. doi: 10.2196/12869 was not cited in the article. The citation has now been inserted in the section **Discussion**, Subsection 4.2 *Digital Health and PHC*, Paragraph 4, and should read: This is supported by a systematic review by Lattie et al. (85) that identified that improving user experience and tailoring user engagement appeared vital for the sustainable implementation of digital mental health interventions.

“low physical inactivity” was used instead of “low physical activity”.

A correction has been made to the section **Introduction**, Paragraph 1, in the published article:

“NCDs such as cardiovascular disease, type-2 diabetes mellitus, chronic respiratory diseases and certain cancers are largely preventable through the elimination of lifestyle risk factors like tobacco use, poor nutrition, low physical activity, and alcohol use (5, 7, 8), making health promotion interventions particularly effective for tackling the global NCD burden.”

“;” was used instead of “:”.

A correction has been made to the section **Materials and Methods**, subsection 2.1 **Setting**, Paragraph 1, in the published article: “Two communities were selected for this study: Athy in Ireland.”

“to” missing from text.

A correction has been made to the section **Materials and Methods**, subsection 2.2 **Ethics**, Paragraph 1, in the published article: “Written informed consent was obtained from all respondents prior to taking part in the survey.”

“for” missing from text.

A correction has been made to the section **Materials and Methods**, subsection 2.3.1 **Quantitative needs assessment survey**, Paragraph 1, in the published article: “This survey also used existing literature on standards of health according to these pillars to provide context for participants completing the assessment. For example, the recommended target for sleep was 7–8 hours per night.”

“km” was used instead of “kilometers”.

A correction needs to be made to the section **Materials and Methods**, subsection 2.4 **Sample and recruitment**, Paragraph 1, line 14 in the published article: “The paid Facebook campaign was restricted to adults over 18.years residing and/or working in Athy and the surrounding hinterland (up to 5 kilometers).”

“the” missing from text.

A correction has been made to the section **Results**, subsection 3.1.2 **Romanian respondents**, Paragraph 1, in the published article: “In terms of the highest level of education attained, 68% of respondents had attended third-level education, 22% attended secondary school, and 10% attended vocational school. Seventy-one percent of respondents were homeowners, 21% of the sample rented, and 6% were living in social housing or emergency accommodation.”

Paragraphs 2 & 3 were the same.

A correction has been made to the section **Results**, subsection 3.2.1 **Athy, Ireland**. Paragraph 2 has been deleted. Paragraph 3 has been retained:

“Barriers to sleep, physical activity, and healthy eating were also assessed (**Supplementary Table S10**). Key barriers to physical activity included low motivation (53%), lack of time due to work/study (41%) or caring responsibilities (18%), in addition to open responses (*n* = 12) citing fatigue (33%) and chronic pain or chronic disease (33%). Suggested solutions (*n* = 101) included safer roads and public spaces (20%), and improved community initiatives and access to exercise groups (10%). For healthy eating, high food costs (40%) and lack of time (40%) were the main barriers, with additional challenges including household food preferences (23%) and limited knowledge (11%). Sleep was most often affected by stress and worry (67%), followed by work (38%) and screen use (30%).”

“and” missing from text.

A correction has been made to the section **Results**, subsection 3.2.2 **Iasi, Romania**, Paragraph 2, in the published article: “Other challenges cited were lack of time (18%), and lack of adapted environments to exercise (16.5%). Suggested solutions included affordable gyms, community-based group activities, and accessible facilities for those with health conditions.”

“for” was used instead of “in”.

A correction has been made to the section **Results**, subsection 3.3.2 **Iasi, Romania**, Paragraph 5, in the published article: “Residents of social or emergency housing and renters reported greater interest in support with sleep hygiene (*p* = 0.046) and mental health (*p* = 0.027).”

“were” was used instead of “was”.

A correction has been made to the section **Results**, subsection 3.4.2 **Iasi, Romania**, Paragraph 1, in the published article: “Engagement was also linked to support, with 76% more likely to use the app if guidance was provided.”

“of” was used instead of “to”.

A correction has been made to the section **Discussion**, Paragraph 3, in the published article: “Similarly, although lifestyle habits and barriers to healthy lifestyle behaviors appeared similar across the two communities, some differences emerged relating to barriers to healthier lifestyles and preferences for support.”

“no” deleted from text.

A correction has been made to the section **Discussion**, Paragraph 4, in the published article: “These findings highlight the pressing need to address gaps in community support systems and the requirement to empower individuals to build a strong foundation for a healthy lifestyle.”

“,” used instead of “,”.

A correction has been made to the section **Discussion**, Subsection 4.1.4 **Residency**, Paragraph1, in the published article: “Similarly, the perceived unpalatability of respondents or their family was a barrier to healthy eating for those living in social housing.”

“has” used instead of “have”.

A correction has been made to the section **Discussion**, Subsection 4.2 **Digital Health and PHC**, Paragraph 2, in the published article: “Results from the scientific literature have indicated that inequity in societies and healthcare systems is usually reflected or exacerbated in digital health applications and use (81–83).”

“a” missing from text.

A correction has been made to the section **Conclusion**, Subsection 6.1 **Implications for future research and practice**, Paragraph 1: “Along with other researchers, we advocate the use of a bottom-up CBPR approach to tailor digital health solutions and reduce health inequities, especially at a community level.”

The original version of this article has been updated.

